# Is alcohol use disorder associated with higher rates of depression and anxiety among people with new onset type 2 diabetes? A cohort study using linked primary care data in England

**DOI:** 10.1186/s12875-024-02628-6

**Published:** 2024-10-30

**Authors:** Sarah Cook, David Osborn, Rohini Mathur, Harriet Forbes, Ravi Parekh, Arti Maini, Ana Luisa Neves, Shamini Gnani, Thomas Beaney, Kate Walters, Sonia Saxena, Jennifer K. Quint

**Affiliations:** 1 School of Public Health, Imperial College London, London, UK; 2 Division of Psychiatry, University College London, London, UK; 3 Camden and Islington NHS Foundation Trust, London, UK; 4 Wolfson Institute of Population Health, Queen Mary University of London, London, UK; 5 Department of Non-Communicable Disease Epidemiology, Faculty of Epidemiology and Population Health, London School of Hygiene and Tropical Medicine, London, UK; 6 Primary Care and Population Health, University College London, London, UK

**Keywords:** Alcohol use disorder, Diabetes, Depression, Anxiety, Electronic health records, General practice

## Abstract

**Introduction:**

Depression and alcohol use disorder (AUD) in people living with Type 2 diabetes mellitus (T2DM) are associated with worse health outcomes. AUD is strongly associated with depression and anxiety, but it is not known how these conditions cluster in people with T2DM. We investigated rates of new episodes of depression and anxiety following T2DM diagnosis in people with and without prior AUD among an English primary care population.

**Methods:**

The study population was people diagnosed with T2DM between 2004 and 2019. We used the Clinical Practice Research Datalink (CPRD) Aurum database and linked Hospital Episode Statistics Admitted Patient Care (HES APC) and Office for National Statistics (ONS) mortality data. We examined incidence of new episodes of anxiety or depression in people with T2DM with and without AUD. AUD was defined as any of i) clinical diagnosis; ii) alcohol withdrawal; or iii) chronic alcohol-related harm (physical or mental) using SNOMED-CT or ICD-10 codes. People were excluded if they had codes for depression/anxiety 12 months prior to T2DM diagnosis. Poisson regression models were fitted adjusting sequentially for a) age, gender, calendar time; b) region, Index of Multiple Deprivation, ethnicity, body mass index, smoking status, Charlson co-morbidity index; and c) history of a mental health condition.

**Results:**

Our study population was 479,447 people of whom 10,983 (2.3%) had an AUD code prior to T2DM diagnosis.

After adjusting for all measured confounders except history of a mental health condition, IRR for depression was 2.00 (95% CI 1.93, 2.06) for people with AUD compared to without AUD. This reduced to 1.45 (95% CI 1.41, 1.50) after further adjustment for history of a mental health condition.

Findings for anxiety were substantially similar to those for depression (adjusted for all measured confounders except history of a mental health condition, IRR 2.08 95% CI 1.99, 2.18 fully adjusted IRR 1.48 95% CI 1.41, 1.55).

**Conclusions:**

People with AUD have over double the rates of depression and anxiety following T2DM diagnosis than those without AUD. This was only partially explained by pre-existing diagnoses of mental health conditions. A holistic approach incorporating mental health support is needed to improve health outcomes for people with AUD who develop T2DM.

**Trial registration:**

Not applicable.

## Background

Diabetes is a serious public health concern ranked as the eighth leading cause of death and disability worldwide in 2019 [1]. Global prevalence of diabetes, particularly type 2 diabetes (T2DM), is increasing [2] with an estimated 529 million people worldwide living with diabetes in 2021 [2]. High levels of alcohol consumption (estimated as > 63 g/day from a meta-analysis from 2015 [3]) have been consistently associated with increased risk of developing type 2 diabetes [3–6].

If blood glucose is not well-controlled, T2DM can have severe consequences including sight loss, amputation, chronic kidney disease, myocardial infarction, and stroke. Management of T2DM is crucial for preventing adverse health outcomes. Management is complex, involving adherence to medication, a healthy diet, physical activity, and attending regular check-ups within primary care. This can put a high burden on patients. Co-occurring mental health conditions including both depression and anxiety are common in T2DM [7–12] and can make management even more challenging.

Depression, anxiety and alcohol use disorder (AUD), an umbrella term for alcohol dependence and alcohol-related harm, are all associated with poorer outcomes from T2DM [13–21]. There is evidence that co-occurring depression and anxiety in people with diabetes are associated with glycemic variability [12, 19, 22], lower treatment adherence [23, 24], poorer quality of life [25], higher health care costs [26], higher risk of complications [13, 15, 21] and mortality [14, 16, 20].

There are high rates of co-occurrence of AUD and common mental disorders (depression and anxiety) in the general population [27–29] but there is little evidence looking at how these conditions cluster together in people with T2DM. Where several conditions are present, this could amplify the effects of each other and increase the risk of adverse outcomes. To our knowledge, no studies have previously investigated whether AUD is associated with an increased risk of developing depression and anxiety following T2DM diagnosis. To target interventions effectively, it is important to quantify to what extent people with AUD are at higher risk of developing depression and anxiety following a diagnosis of T2DM.

The aim of this paper was to investigate if people with AUD were more likely to develop a new episode of depression or anxiety following T2DM diagnosis compared to those without AUD among a primary care population in England.

## Methods

### Study sample and data source

We used data from the Clinical Practice Research Datalink (CPRD) Aurum [30, 31]. CPRD Aurum contains electronic health record (EHR) data from a sample of primary care practices within the UK that use EMIS Web® general practice patient management software, collected during routine healthcare. This includes information on demographic characteristics, diagnoses and symptoms, medication prescriptions and laboratory tests. The CPRD Aurum population is representative of the UK population in terms of age, gender, geographic location and deprivation [30]. The May 2022 version of the database used for this study covered 19.83% of the UK population [31].

CPRD Aurum data were linked with Hospital Episode Statistics Admitted Patient Care Data (HES APC) [32], and Index of Multiple Deprivation (IMD) [33].

HES APC contains data on patients admitted to National Health Service (NHS) hospitals in England. As eligibility for linkage with HES data was a requirement for this study, we restricted our study population to people resident in England rather than the whole UK population.

### Cohort construction and case definition

The target population was people with incident T2DM between 1st January 2004 and 1^st^ January 2020. We restricted our study period to pre 2020 to avoid biasing incidence estimates from health service disruption during the COVID-19 pandemic [34].

We defined date of T2DM incidence as the first date for a T2DM diagnosis or a T2DM -related code from a broader code list of diabetes SNOMED CT codes (https://github.com/NHLI-Respiratory-Epi/diabetes_alcohol_use_disorder/blob/main/diabetes_broad_list_vs3.xls).

**Fig. 1:**
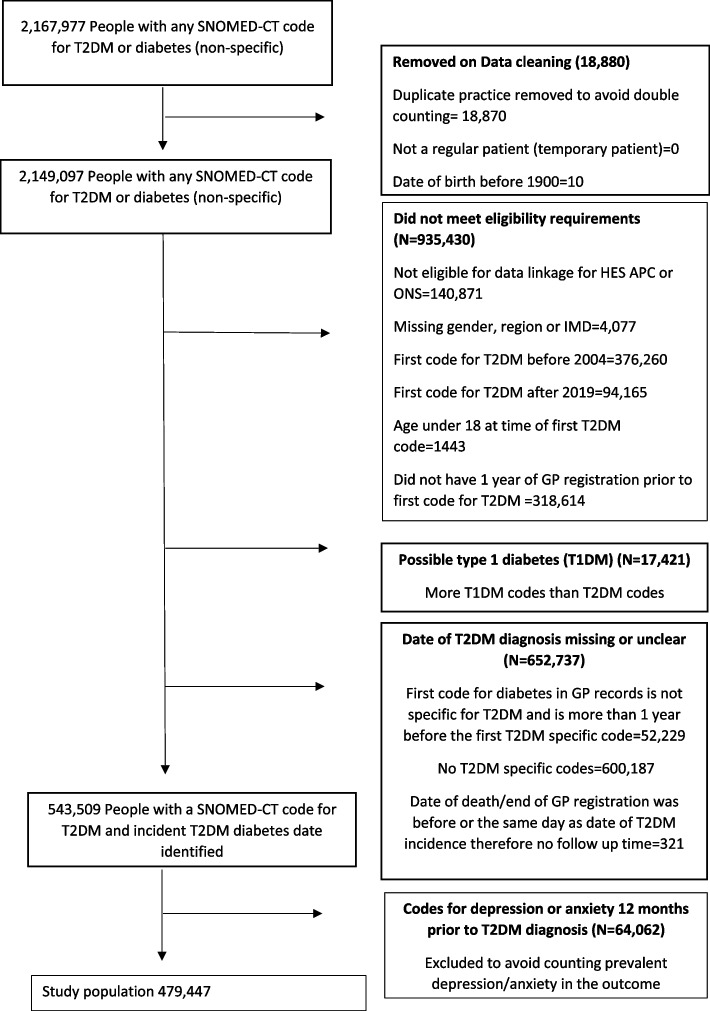
Selection of People with a diagnosis of type 2 diabetes between 2004 and 2019

We excluded people if they were aged less than 18 at the time of diagnosis, their gender, region or IMD was missing, they were not a fully registered patient; were not eligible for linkage to HES APC and IMD datasets, or were registered in a GP practice outside of England. To capture incident (not prevalent) T2DM, we also excluded people if they had less than 1 year of registration prior to their first T2DM code; their first code for diabetes was not diagnostic for T2DM and was more than a year from the time of first T2DM code. We also excluded people if they did not have any codes specific to T2DM (only codes for diabetes in general without specifying type) or if they had more type 1 diabetes (T1DM) codes than T2DM codes (Fig. 1). Codes for gestational diabetes were not classed as incident T2DM but if someone had codes for gestational diabetes and codes for T2DM then their first T2DM codes was classed as the date of T2DM diagnosis.

### Alcohol use disorder

Alcohol use disorder was defined from SNOMED-CT codes entered by health care practitioners within primary care or ICD-10 codes from hospital admitted patient care records.

For the SNOMED-CT codes we adapted the International Classification of Disease 10 (ICD-10) definition of AUD to capture diagnoses of alcohol dependence syndrome and harmful alcohol use as defined in Chapter F10.1-F10.9 Mental and Behavioural disorders due to Alcohol [35]. The codes used were categorised as AUD independently by at least two of the co-authors (SC reviewed all codes and TB, SG, ALN, AM, RP (all practicing general practitioners within primary care in England) who each reviewed a subset of codes (100–200 codes each)). Divergent codes were discussed by at least three of the co-authors and a consensus reached.

In line with this definition, AUD was defined as presence of a SNOMED-CT code in the primary care record which indicated i) an AUD diagnosis in line with ICD-10 definitions or ii) alcohol withdrawal as a symptom of alcohol dependence syndrome or iii) chronic alcohol-related harm to health (physical or mental) in line with an ICD-10 diagnosis of harmful alcohol use; or any ICD-10 code from F10.1 – F10.9 from within hospital inpatient records prior to the date of T2DM diagnosis.

### Outcome definition

The outcomes were new episodes of depression or anxiety following diagnosis of T2DM. Depression and anxiety were defined from SNOMED-CT codes entered by health care practitioners within primary care or ICD-10 codes from hospital admitted patient care records.

New episode depression was classified as the earliest of i) any SNOMED-CT code indicating definite or probable depression or ii) a SNOMED-CT code indicating possible depression plus a prescription for an antidepressant medication within 3 months or iii) any ICD 10 code from hospital inpatient records indicating depression (Any codes within chapter F32(depressive episode); F33 (recurrent depressive episode); F34 (persistent mood disorders); F41.2 (mixed anxiety and depressive disorder) and F92.0 Depressive conduct disorder).

Definite/probable depression refers to SNOMED-CT codes which were more specific for depression, for example a diagnostic code. Possible depression refers to codes which were less specific, for example depression symptoms or use of screening tools for depression. SNOMED-CT codes related to the following diagnoses were included: major depressive disorder, dysthymia, recurrent depressive disorder, seasonal affective disorder, mixed anxiety and depression, disruptive mood dysregulation disorder, depression in dementia (or other condition), trauma and stress-related disorders with depressed mood, including adjustment disorders with depressed mood.

The same definition was used for anxiety but for equivalent codes for anxiety and anxiolytic medication. For anxiety from hospital episode statistics data we used the following ICD-10 codes: any codes within F40 (phobic anxiety disorders); F41 (other anxiety disorders); F42 (obsessive–compulsive disorder); F43 (reaction to severe stress and adjustment disorders); F44 (Dissociative [conversion] disorder) and F48 (other neurotic disorders). SNOMED-CT codes related to the following diagnoses were included: generalized anxiety disorder, panic disorder, mixed anxiety and depression, obsessive compulsive disorders, trauma- and stress-related disorders with anxiety, anxiety disorder NOS, agoraphobia, and social anxiety disorder.

People with codes for depression or anxiety in the 12 months prior to T2DM diagnosis using the same definitions as for the study outcome were excluded from analyses to prevent existing episodes of depression/anxiety at the time of diagnosis being treated as new episodes.

All codelists for depression and anxiety are available on Github (https://github.com/NHLI-Respiratory-Epi/diabetes_alcohol_use_disorder).

### Confounding variables

Confounding variables included are listed in Table 1.

**Table 1:** Confounding factors included in the model

• Age
• Gender
• Calendar time (year of T2DM diagnosis and calendar period)
• Region
• Ethnicity (White, Asian, Black, Mixed and Other)
• Quintiles of area-level socio-economic status measured by the Index of Multiple Deprivation
• Most recent smoking status
• Most recent body mass index (BMI)
• Prior history of physical health conditions defined as number of co-morbidities using the Charlson Co-morbidity Index [36] excluding diabetes
• Prior diagnoses of mental health conditions

Prior history of diagnosed mental health conditions (depression for anxiety analyses, anxiety for depression analyses, severe mental illness (SMI)) was considered in analyses as a potential confounder, but was also considered as potential mediator, and models were included with and without adjustment for prior diagnoses of mental health conditions.

SMI was defined as either a SNOMED-CT code within primary care or any ICD 10 code in inpatient hospital records at any time point prior to T2DM diagnosis for schizophrenia, other psychotic disorders or bipolar disorder.

Ethnicity, smoking and BMI were defined using previously described algorithms [37, 38].

All other variables were defined using primary care data using codelists available on Github (https://github.com/NHLI-Respiratory-Epi/diabetes_alcohol_use_disorder).

### Statistical analysis

The baseline characteristics of people with and without AUD codes recorded at the time of T2DM diagnosis were compared using proportions for categorical variables and mean and standard deviation or median and interquartile range for continuous variables. Kaplan–Meier curves were plotted showing rates of new episode depression and anxiety from the time of T2DM diagnosis stratified by history of past depression and anxiety.

Poisson regression models were fitted separately for outcomes a) new episode depression and b) new episode anxiety with AUD at the time of T2DM diagnosis as the exposure adjusting sequentially for i) age at T2DM diagnosis, gender, and year of T2DM diagnosis, current age and calendar time, ii) region, IMD, ethnicity, smoking status, body mass index, and physical co-morbidities at the time of T2DM diagnosis measured using the Charlson Co-morbidity Index and iii) past history of depression, anxiety and SMI. Sequential adjustment was conducted to explore first the association with minimal adjustment (model 1), with adjustment for all measured confounders (model 2) and thirdly with additional adjustment for past mental health history to understand to what extent past mental health history explained any observed associations (model 3). Study follow up for each person started from date of T2DM diagnosis and ended at the earliest of first episode of the outcome (depression or anxiety respectively), death, end of GP registration, last collection date of the GP practice or 1st January 2020. Data were split into ten year age bands and 4 year calendar periods and current age and calendar period were adjusted for within all models with the assumption that incidence rates were constant within these age and time bands.

Interactions investigated were i) calendar period to test whether effect estimates were constant over calendar time and ii) history of past depression (where depression was the outcome) and iii) history of past anxiety (where anxiety was the outcome) to test whether there was effect modification by previous history of depression/anxiety. Interactions were investigated by fitting the same Poisson models as for main analyses with and without interaction terms between the interaction variable of interest (calendar period/history of past depression/history of past anxiety respectively) and AUD and comparing models with and without interaction terms using likelihood ratio tests.

We hypothesized rates would be different for people who were currently drinking compared to people who were not currently drinking at the time of their T2DM diagnosis. Therefore, we also investigated the impact of dividing the AUD group into people who were more likely to be drinking at the time of their diagnosis of T2DM and people with evidence to suggest they were not currently drinking at the time of the T2DM diagnosis. The “not currently drinking” group was defined from 1) a SNOMED-CT code within primary care for non-drinking more recent than their most recent code for AUD or 2) most recent AUD SNOMED-CT code in primary care indicated remission or 3) no AUD codes in primary or secondary care in the past 5 years.

Complete case analysis was used for regression modelling restricting models to people with complete data on all variables.

Analyses were conducted using Stata 17.

## Results

There were 479,447 people in the sample with an incident diagnosis of T2DM between 1^st^ January 2004 and 31^st^ December 2019 and no codes for depression or anxiety in the year before T2DM diagnosis. Of these, 10,983 (2.3%) were coded as having AUD prior to diagnosis of T2DM.

The characteristics of the study population at time of T2DM diagnosis are shown in Table 2. People with a code for AUD prior to T2DM diagnosis had a higher prevalence of pre-existing mental health conditions compared to people without AUD codes prior to T2DM diagnosis (depression 43.8% vs 20.1%; anxiety 28.8% vs 13.0% and SMI 9.2% vs 2.0%; *p* < 0.001 for all three conditions) (Table 2).

**Table 2:** Characteristics of people with and without a code for Alcohol Use Disorder (AUD) prior to Type 2 Diabetes diagnosis at time of type 2 diabetes (T2DM) diagnosis^a^

		AUD code recorded prior to type 2 diabetes diagnosis		No AUD code prior to type 2 diabetes diagnosis		Total study population	
		N	(%)	N	(%)	N	(%)
Total		10,983	(100)	468,464	(100)	478,447	(100)
Age at time of T2DM diagnosis	Mean (Standard Deviation)	58.4	(11.5)	61.6	(13.9)	61.5	(13.9)
Gender	Male	8,594	(78.3)	266,098	(56.8)	274,692	(57.3)
	Female	2,389	(21.8)	202,366	(43.2)	204,755	(42.7)
Ethnicity	White	9,902	(90.9)	372,362	(81.0)	382,264	(81.2)
	Asian	528	(4.9)	53,801	(11.7)	54,329	(11.5)
	Black	309	(2.8)	24,194	(5.3)	24,503	(5.2)
	Mixed	79	(0.7)	4,616	(1.0)	4,695	(1.0)
	Other	79	(0.7)	4,884	(1.1)	4,963	(1.1)
	Not stated/ missing (% of cohort)	86	(0.8)	8,607	(1.8)	8,693	(1.8)
Region of England	North East England	508	(4.6)	16,505	(3.5)	17,013	(3.6)
	North West England	2,665	(24.3)	86,728	(18.5)	89,393	(18.7)
	Yorkshire and the Humber	395	(3.6)	16,811	(3.6)	17,206	(3.6)
	East Midlands	233	(2.1)	11,092	(2.4)	11,325	(2.4)
	West Midlands	1,653	(15.1)	86,316	(18.4)	87,969	(18.4)
	East of England	387	(3.2)	19,606	(4.2)	19,993	(4.2)
	London	1,981	(18.0)	89,613	(19.1)	91,594	(19.1)
	South East England	1,740	(15.8)	86,342	(18.4)	88,082	(18.4)
	South West England	1,421	(12.9)	55,451	(11.8)	56,872	(11.9)
Index of multiple deprivation	1 – Least deprived	1,313	(12.0)	82,477	(17.6)	83,790	(17.5)
	2	1,665	(15.2)	89,518	(19.1)	91,183	(19.0)
	3	1,940	(17.7)	91,197	(19.5)	93,137	(19.4)
	4	2,567	(23.4)	101,430	(21.7)	103,997	(21.7)
	5 – Most deprived	3,498	(31.9)	103,842	(22.2)	107,340	(22.4)
Year of diagnosis of T2DM	Median (IQR)	2013	(2009, 2016)	2012	(2008,2016)	2012	(2008, 2016)
Most recent recorded smoking status	Never	973	(9.0)	103,138	(22.4)	104,111	(22.1)
	Ex	5,283	(48.8)	282,672	(61.3)	287,955	(61.0)
	Current	4,578	(42.3)	75,488	(16.2)	80,066	(17.0)
	Missing (*% of cohort)	149	(1.4)	7,166	(1.5)	7,315	(1.5)
Body Mass Index	Underweight (< 18.5)	158	(1.5)	1,613	(0.4)	1,771	(0.4)
	Normal (18.5–24.9)	1,585	(15.4)	48,298	(11.0)	49,883	(11.1)
	Overweight (25–29.9)	3,110	(30.3)	139,229	(31.8)	142,339	(31.7)
	Obese (≥ 30)	5,422	(52.8)	249,148	(56.9)	254,570	(56.8)
	Missing (*% of cohort)	708	(6.5)	30,176	(6.4)	30,844	(6.4)
Past depression	Yes	4,805	(43.8)	94,007	(20.1)	98,812	(20.6)
Past anxiety	Yes	3,159	(28.8)	60,959	(13.0)	64,118	(13.4)
Severe mental illness	Yes	1,015	(9.2)	9,357	(2.0)	10,372	(2.2)
Number of co-morbidities according to Charlson Co-morbidity Index (excluding diabetes)	0	4,249	(38.7)	249,729	(53.3)	253,978	(53.0)
	1	3,795	(34.6)	140,994	(30.1)	144,789	(30.2)
	2	1,869	(17.0)	51,557	(11.0)	53,426	(11.1)
	3 +	1070	(9.7)	26,184	(5.6)	27,254	(5.7)
Current drinking at the time of diagnosis in people with AUD	Not currently drinking	3,815	(34.7)	-		-	
	Currently drinking	7,168	(65.3)	-		-	
Source of diagnosis for AUD	Primary care only	4804	(43.7)	-		-	
	Hospital inpatient record only	3,512	(32.0)	-		-	
	Both hospital and primary care record	2,667	(24.3)	-		-	

^a^People with codes for depression or anxiety in the past 12 month prior to T2DM diagnosis excluded

### Association between AUD and incident depression following diagnosis of T2DM

For depression analyses follow up time was 2,256,586 person years for people without codes for AUD and 34,973 person years for people with codes for AUD. Median follow up time was 3.7 years (IQR 1.40–7.29 years).The rate of incident depression was 123.9 (95% CI 120.3, 127.6) per 1000 person years in people with a code for AUD compared to 51.6 (95% CI 51.3, 51.9) per 1000 person years in people with no codes for AUD, a rate difference of 72.3 per 1000 person years (Table 3).

**Table 3:** Rates of new episode depression following type 2 diabetes (T2DM) diagnosis by whether people had a code for alcohol use disorder (AUD) at the time of T2DM diagnosis and history of previous depression

		Events	Rate per 1000 person years (95% CI)	Rate Difference (People with AUD- people without AUD)
Total sample	No AUD code	116,485	51.6 (51.3, 51.9)	72.3
	AUD code	4333	123.9 (120.3, 127.6)	
No history of depression	No AUD code	69,133	34.9 (34.6, 35.1)	26.8
	AUD code	1541	61.7 (58.7, 64.8)	
History of depression prior to T2DM diagnosis	No AUD code	47,352	173.1 (171.6, 174.7)	106.4
	AUD code	2,792	279.5 (269.3, 290.0)	

After minimal adjustment (Model 1), people with AUD had an incidence rate ratio (IRR) of depression 2.44 times higher than people without AUD (95% CI 2.37, 2.52). After adjusting for measured confounders except past history of mental health conditions (Model 2) this reduced to 2.00 (95% CI 1.93, 2.06). Further adjustment for past history of mental health conditions partially explained this association (Model 3), however people with AUD still had almost 50% higher rate of depression (IRR 1.45 95% CI 1.41, 1.50) even after adjusting for past history of depression, anxiety and SMI (Table 4).

**Table 4:** Incidence rate ratio for incident depression following diagnosis of Type 2 Diabetes (T2DM) by whether people have an Alcohol Use Disorder (AUD) code in their medical record prior to diagnosis

*N* = 439,494^a^		Model 1^b^		Model 2^c^		Model 3^d^		*P* value for difference model 3
		Incidence rate ratio	95% CI	Incidence rate ratio	95% CI	Incidence rate ratio	95% CI	
Total sample		2.44	2.37, 2.52	2.00	1.93, 2.06	1.45	1.41, 1.50	
Stratified by calendar time^e^	2004–2007	2.53	2.31, 2.77	2.11	2.04, 2.18	1.50	1.45, 1.55	*P* = 0.91
	2008–2011	2.50	2.35, 2.67	2.04	1.92, 2.18	1.50	1.41, 1.60	
	2012–2015	2.62	2.46, 2.79	2.13	2.00, 2.27	1.52	1.43, 1.62	
	2016–2019	2.69	2.56, 2.84	2.16	2.05, 2.28	1.49	1.42, 1.57	
Stratified by history of depression	No history of depression	1.81	1.71, 1.91	1.60	1.52, 1.69	1.56^f^	1.48, 1.64	*P* = 0.002
	History of depression	1.66	1.59, 1.73	1.48	1.42, 1.54	1.40^f^	1.34, 1.46	
AUD stratified by likely drinking status at time of T2DM diagnosis	Not currently drinking	2.54	2.41, 2.68	2.07	1.96, 2.18	1.36	1.29, 1.44	*P* = 0.003
	Currently drinking	2.39	2.30, 2.49	1.96	1.88, 2.03	1.51	1.45, 1.57	

^a^Total sample with no missing data on any variables within the model

^b^Sex, age and year of T2D diagnosis, current age and calendar time

^c^Model 1 + region, IMD quintile, ethnicity, BMI, smoking status, number of co-morbidities from Charlson co-morbidity index

^d^Model 2 + past history of depression, past history of anxiety, past history of severe mental illness

^e^Not adjusted for calendar year of T2DM diagnosis to avoid over adjustment

^f^Not adjusted for past history of depression to avoid over adjustment

**Fig. 2:**
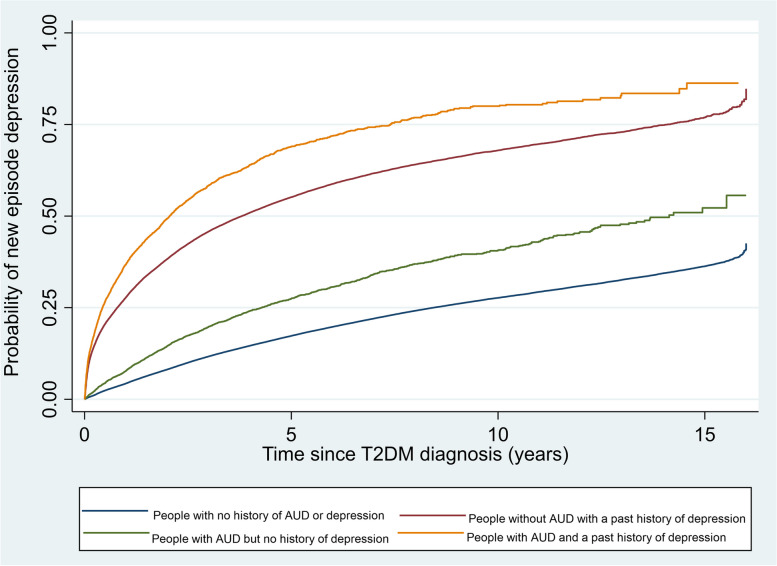
Kaplan–Meier graph of time to new episode of depression post Type 2 Diabetes (T2DM) diagnosis by Alcohol Use Disorder (AUD) and history of depression

**Fig. 3:**
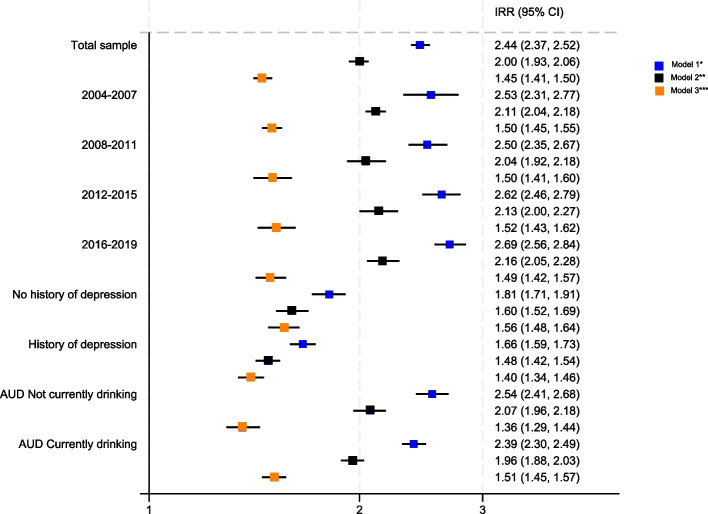
Incidence rate ratio for incident depression following diagnosis of Type 2 Diabetes (T2DM) by whether people have an Alcohol Use Disorder (AUD) code in their medical record prior to diagnosis Figure Legend: *Model 1 adjusted for sex, age and year of T2D diagnosis, current age and calendar time. **Model 2 = Model 1 + region, IMD quintile, ethnicity, BMI, smoking status, number of co-morbidities from Charlson co-morbidity index. ***Model 3 = Model 2 + past history of depression, past history of anxiety, past history of severe mental illness

The absolute rate of incident depression was highest in people with both a history of depression and AUD (Fig. 2 and Table 4). However, while the rate difference in rates of depression by whether people had AUD or not was higher in people with a past history of depression (106.4 per 1000) compared to those without a past history of depression (26.8 per 1000) (Table 3), after adjusting for all measured confounders including history of anxiety and SMI (model 3), there was a statistical evidence for interaction in the opposite direction i.e. the IRR was higher in people with no prior codes for depression (IRR 1.56 95% CI 1.48, 1.64) compared to people with codes for a history of depression (IRR 1.40 95% CI 1.34, 1.46) (test for interaction *p* = 0.002) (Table 4 and Fig. 3).

When the AUD groups were stratified by likely drinking status at T2DM diagnosis, the fully adjusted IRR (Model 3) was slightly higher for people AUD who were likely to be currently drinking (IRR 1.51 95% CI 1.45, 1.57) than for people with AUD who were likely not currently drinking (IRR 1.36 95% CI 1.29, 1.44) *p* value for a difference = 0.003 (Table 4 and Fig. 3).

There was no evidence that the association between AUD and depression was changing with calendar period (*p* = 0.91) (Table 4).

### Association between AUD and incident anxiety following diagnosis of T2DM

For anxiety analyses, follow up time was 2,660,611 person years for people without codes for AUD and 47,140 person years for people with codes for AUD. Median follow up time was 4.7 years (IQR 1.99–8.69 years).The rate of incident anxiety was 45.3 (95% CI 43.3, 47.3) cases per 1000 person years in people with a code for AUD and 26.4 (95% CI 24.8, 25.2) cases per 1000 person years in people with no code for AUD, a rate difference of 26.4 per 1000 person years (Table 5).

**Table 5:** Rates of new episode anxiety following type 2 diabetes (T2DM) diagnosis by whether people had a code for alcohol use disorder (AUD) at the time of T2DM diagnosis and history of previous anxiety

		Events	Rate per 1000 person years	Rate Difference (People with AUD- people without AUD)
Total sample	No AUD code	50,223	18.9 (18.7, 19.0)	26.4
	AUD code	2,139	45.3 (43.4, 47.3)	
No history of anxiety	No AUD code	32,642	13.7 (13.5, 13.8)	12.9
	AUD code	964	26.6 (24.9, 28.3)	
History of anxiety prior to T2DM diagnosis	No AUD code	17,581	64.9 (64.0, 65.9)	42.9
	AUD code	1,175	107.8 (101.8,114.1)	

Substantive findings for the association between AUD and incident anxiety were very similar to those for depression.

After minimal adjustment (Model 1), people with AUD had an incidence rate ratio (IRR) of anxiety 2.56 times higher than people without AUD (95% CI 2.45, 2.68). After adjusting for measured confounders except past history of mental health conditions (Model 2), this reduced to 2.08 (95% CI 1.99, 2.18). As for depression, further adjustment for past history of mental health conditions partially explained this association (Model 3), however people with AUD still had almost 50% higher rates of anxiety (IRR 1.48 95% CI 1.41, 1.55) even after adjusting for past history of depression, anxiety and SMI (Table 6).

**Table 6:** Incidence rate ratio for incident anxiety following diagnosis of Type 2 Diabetes (T2DM) by whether people have an Alcohol Use Disorder (AUD) code in their medical record prior to diagnosis

*N* = 439,494^a^		Model 1^b^		Model 2^c^		Model 3^d^		*P* value for difference model 3
		Incidence rate ratio	95% CI	Incidence rate ratio	95% CI	Incidence rate ratio	95% CI	
Total sample		2.56	2.45, 2.68	2.08	1.99, 2.18	1.48	1.41, 1.55	
Stratified by Calendar time^e^	2004–2007	2.34	1.92, 2.87	1.91	1.56, 2.33	1.36	1.11, 1.67	*P* = 0.38
	2008–2011	2.75	2.45, 3.08	2.24	2.00, 2.52	1.58	1.40, 1.77	
	2012–2015	2.52	2.30, 2.77	2.05	1.87, 2.25	1.43	1.30, 1.57	
	2016–2019	2.74	2.59, 2.91	2.21	2.08, 2.35	1.53	1.44, 1.63	
Stratified by history of anxiety	No history of anxiety	2.13	1.99, 2.28	1.62	1.52, 1.74	1.60^f^	1.50, 1.72	*P* = 0.001
	History of anxiety	1.74	1.64, 1.85	1.41	1.33, 1.50	1.38^f^	1.30, 1.47	
AUD stratified by drinking status at time of T2DM diagnosis	Not currently drinking	2.51	2.33, 2.71	2.04	1.89, 2.20	1.33	1.24, 1.44	*P* = 0.001
	Currently drinking	2.59	2.45, 2.73	2.10	1.99, 2.22	1.56	1.48, 1.65	

^a^Total sample with no missing data on any variables within the model

^b^Sex, age & year of T2D diagnosis, current age and calendar time

^c^Model 1 + region, IMD quintile, ethnicity, BMI, smoking status, number of co-morbidities from Charlson co-morbidity index

^d^Model 2 + past history of depression, past history of anxiety, past history of severe mental illness

^e^Not adjusted for calendar year of T2DM diagnosis to avoid over adjustment

^f^Not adjusted for past history of anxiety to avoid over adjustment

**Fig. 4:**
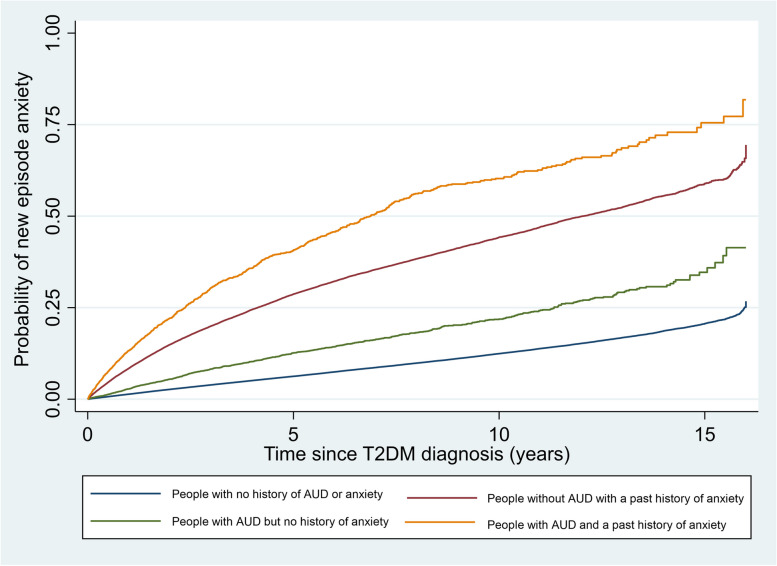
Kaplan–Meier graph of time to new episode anxiety post Type 2 Diabetes (T2DM) diagnosis by Alcohol Use Disorder (AUD) and past anxiety

**Fig. 5:**
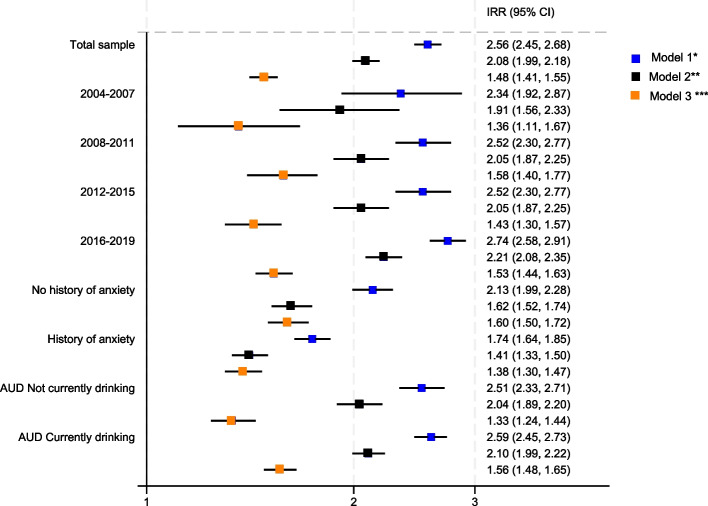
Incidence rate ratio for incident anxiety following diagnosis of Type 2 Diabetes (T2DM) by whether people have an Alcohol Use Disorder (AUD) code in their medical record prior to diagnosis. Figure Legend: *Model 1 adjusted for sex, age and year of T2D diagnosis, current age and calendar time. **Model 2 = Model 1 + region, IMD quintile, ethnicity, BMI, smoking status, number of co-morbidities from Charlson co-morbidity index. ***Model 3 = Model 2 + past history of depression, past history of anxiety, past history of severe mental illness

The absolute rate of incident anxiety was highest in people with both a history of anxiety and AUD (Fig. 4 and Table 5). However, while the rate difference in rates of anxiety by whether people had AUD or not was higher in people with a past history of anxiety (42.9 per 1000) compared to those without a past history of anxiety (12.9 per 1000) (Table 3), after adjusting for all measured confounders (model 3) there was a statistical evidence that the IRR was higher in people with no prior codes for anxiety(IRR 1.60 95% CI 1.50, 1.72) compared to people with codes for a history of anxiety (IRR 1.38 95% CI 1.30, 1.47) (*p* = 0.001) (Table 6 and Fig. 5).

When the AUD groups were stratified by likely drinking status at T2DM diagnosis, the fully adjusted IRR (Model 3) was slightly higher for people with AUD who were likely to be currently drinking (IRR 1.56 95% CI 1.48, 1.65) than for people with AUD who were likely not currently drinking (IRR 1.33 95% CI 1.24, 1.44) *p* value for a difference *p* = 0.001 (Table 6 and Fig. 5).

There was no evidence that that association between AUD and anxiety changed over calendar period (*p* = 0.38) (Table 6 and Fig. 5).

## Discussion

In this study of people with T2DM and AUD identified from electronic health care records, people with AUD had approximately two and a half times higher rates of developing a new episode of depression or anxiety. While a history of mental health problems at the time of T2DM diagnosis was a strong factor explaining this effect, adjusting for this did not explain this completely. People with AUD were still approximately 50% more likely to be diagnosed with depression or anxiety after a diagnosis of T2DM, even after adjusting for measured confounders including history of depression, anxiety and SMI. Our findings were consistent over calendar time and applied to people with and without a prior history of depression/anxiety.

There was strong evidence that incidence rate ratios for depression and anxiety were higher in people with AUD who were likely to be currently drinking compared to not currently drinking, after accounting for all measured confounders, including past medical history. However, those who were categorized as likely to be not currently drinking still had 36% higher rate of depression and 33% higher rates of anxiety than people without AUD, suggesting ever having an AUD if associated with longer-term effects on mental health even if someone is not currently drinking. Our AUD definition included chronic alcohol-related harms to physical and mental health, which may contribute to the continued impact of AUD on mental health even among people who were likely to be not drinking at the time of their T2DM diagnosis.

Current UK guidance from National Institute of Health and Clinical Excellence (NICE) is to treat AUD first before treating depression or anxiety in the presence of co-occurring AUD and depression or anxiety symptoms [39]. This may mean our findings underestimate the true effect of AUD on incidence of depression and anxiety, particularly in those who are currently drinking, as GPs may be less likely to diagnose depression or anxiety in people with AUD who are currently drinking.

Despite this possible under-ascertainment, we found people with a history of AUD were at substantially higher risk of developing both anxiety and depression following diagnosis of T2DM than people without a history of AUD. The increased rates of incident depression in people with AUDfound here is important both for the negative impact on quality of life [25] but also for the potential negative impact on diabetes prognosis. Co-morbid depression and anxiety in people with diabetes are associated with glycemic variability [12, 19, 22], lower treatment adherence [23, 24], higher risk of complications [13, 15, 21] and death [14, 16, 20]. From a clinical perspective, our study highlights the importance of support for people with a history of AUD who develop T2DM, both to stop drinking if they are currently drinking, and also with their mental health more generally.

### Comparison with previous literature

A previous study using EHR data from the US found that among 16,243 people with prevalent T2DM and substance use disorder had 2.22 times higher odds of a prevalent mood disorder and 1.87 times higher odds of anxiety than people without substance use disorder after adjusting for demographic factors and other physical co-morbidities. However, this study was cross-sectional in nature and did not distinguish between alcohol use from tobacco and other drug use [40]. To our knowledge, our study is the first to investigate longitudinally whether AUD is associated with higher incidence of depression and anxiety following diagnosis of T2DM.

A systematic review and meta-analysis found AUD was associated with a risk ratio of 1.57 (with a range from 1.03–2.43) for incident depression among study samples not restricted to people with T2DM [29]. While it is hard to make a direct comparison, given the 16 studies included in the meta-analysis adjusted for different confounders to each other and to our study, the highest effect estimate in the meta-analysis (2.43) is from a population-based cohort study of 3481 adults in East Baltimore in the US which adjusted for age, sex and years of education only [41]. This is very similar to our minimally adjusted IRR (2.44), while the pooled risk ratio from the meta-analysis was close in size to our fully adjusted IRR (1.45). Overall, this suggests that the association of AUD with incident depression was similar in our sample of people with T2DM, with similar increased rates of depression in people with AUD compared to without AUD to studies from the general population, and the co-occurrence of T2DM does not seem to amplifying further the association between AUD and depression. Effect estimates from a longitudinal study population-based study from the Netherlands investigating the association between AUD and anxiety disorder at three year intervals among 6,646 participants were also of a similar effect size to our study after adjusting for socio-demographic factors, smoking and clinical factors (odds ratio for prediction of prevalent anxiety disorder 1.69 95% CI 1.11–2.43; odds ratio for first episode anxiety 2.03 95% CI 1.17–3.51). However the study design (data collected at three year intervals) and effect estimates from this study are not the same (odds ratios compared to incident rate ratio) limiting direct comparisons between the studies [42]. Further work comparing our findings in comparable study populations with and without T2DM is needed to confirm this.

### Strengths and limitations

CPRD Aurum is a large nationally representative database. Although AUD is a relatively rare condition, we were able to identify 10,983 people meeting the study criteria for AUD with incident T2DM, providing sufficient power to investigate the research question of interest.

EHR data are routinely collected data and are not collected for research purposes. Measurement error is always a consideration when using these data. Completeness and quality of primary care data is dependent both on the coding practices by GPs when recording what takes place during a consultation and on people being in contact with health services. There will be variation between individual GPs and GP practices in how coding is conducted. There may have been misclassification of the exposure and outcome. In our study population AUD was determined from the presence of codes within primary care and hospital records. This will not include people who did not have a diagnosis of AUD recorded within primary care or in their hospital records due to lack of health service contact or under-recognition. Similarly, people who do not seek help for depression and anxiety or who did not have this coded will be misclassified as not developing the outcome. This may have led to bias in the findings if people with AUD are more or less likely to seek medical help for their depression and anxiety symptoms or to receive a diagnosis/code, for example clinicians may be less likely to give a diagnosis of depression or anxiety in people with AUD who are currently drinking due to current clinical guidance on treating the AUD first [39]. This may have led to an underestimate of the true rates of depression and anxiety in people with AUD. While we have used codelists which were reviewed by clinicians to mitigate measurement error it was beyond the scope of this study to formally validate these and there is the potential for misclassification of AUD, depression and anxiety. Furthermore only information recorded in the form of SNOMED-CT or ICD-10 codes was available for research purposes—information recorded as free text was not available for data protection purposes. This increases the potential for misclassification if GPs recorded information on mental health or alcohol use in free text but did not record an associated code in the EHR record.

Here we have considered AUD as a binary exposure at the time of T2DM diagnosis and have not considered alcohol use overall or variations in drinking pattern. We have also not captured changes in drinking over time. While we tried to capture whether people with AUD were likely to be currently drinking or not at the time of T2DM diagnosis, this was defined pragmatically based on what information was captured within the EHR data and misclassification for some people is likely. Residual confounding by unmeasured or incompletely recorded confounders is also a limitation when using these data. For example, there were missing data for some of the confounders (BMI, smoking status and ethnicity) and we were only able to adjust for area level and not individual socio-economic status. The same issues around potential misclassification and measurement are true for confounders as for depression, anxiety and AUD and even where present, smoking or BMI data may not be contemporary or accurate. In addition only smoking status and not a measure of amount smoked for example pack years was included. There are also many other factors like education, employment status, social networks and traumatic life events which may influence both AUD and depression and anxiety. However, these are not recorded in EHRs and therefore could not be adjusted for.

### Public health relevance and future work

We have identified here that people with AUD are at higher risk of depression and anxiety following a diagnosis of T2DM than people without AUD. As well as the strong negative impact on people’s quality of life in terms of the distress caused by depression and anxiety, this may also lead to worse physical health outcomes, given existing evidence base showing depression and anxiety in diabetes are associated with poorer outcomes.

People with AUD are at high risk of developing multiple long term conditions [43] with negative impacts on their health and well-being. The magnitude of effect size for the association between AUD and depression was similar in our study to studies from the general population high-lighting the need for mental health support for all people with AUD regardless of whether they have T2DM. However given the additional complexities of living with T2DM future work is needed to understand whether more targeted interventions would better support the mental health needs of people with AUD and T2DM. This should include strategies for increasing integrated and holistic care which consider the specific issues for people living with both AUD and T2DM. Options should be explored for how best to do this within the range of services supporting people with AUD and T2DM including but not limited to primary care, alcohol recovery services, and peer support groups for both conditions. This study has shown in particular the importance for future work in this area of including mental health support as an integral component of care for people with AUD and T2DM.

## Conclusions

Among people with T2DM, people with AUD have over double the rates of depression and anxiety following their diabetes diagnosis compared to those without AUD. This was only partially explained by pre-existing diagnoses of mental health conditions. A holistic approach incorporating mental health support is needed to improve health outcomes for people with AUD who develop T2DM.

## Data Availability

Data are available on request from the CPRD. Their provision requires the purchase of a license, and this license does not permit the authors to make them publicly available to all. This work used data from the version collected in May 2022 and have clearly specified the data selected within each Methods section. To allow identical data to be obtained by others, via the purchase of a license, the code lists will be provided upon request. Licenses are available from the CPRD (http://www.cprd.com): The Clinical Practice Research Datalink Group, The Medicines and Healthcare products Regulatory Agency, 10 South Colonnade, Canary Wharf, London E14 4PU.

## Abbreviations

AUD: Alcohol use disorder
BMI: Body mass index
CI: Confidence interval
CPRD: Clinical practice research datalink
HER: Electronic health record
GP: General practice/general practitioner
HES APC: Hospital episode statistics admitted patient care
ICD-10: International classification of disease 10
IMD: Index of multiple deprivation
IRR: Incidence rate ratio
NHS: National Health Service
NICE: National Institute of Health and Clinical Excellence
ONS: Office for National Statistics
SMI: Severe mental illness
T2DM: Type 2 Diabetes Mellitus
UK: United Kingdom
US: United States
